# A review of ocular adverse events of biological anti-TNF drugs

**DOI:** 10.1186/s12348-020-00202-6

**Published:** 2020-04-27

**Authors:** Fernanda Nicolela Susanna, Carlos Pavesio

**Affiliations:** 1 Medicine School, University of São Paulo, São Paulo, Brazil; 2 Moorfields Eye Hospital NHS Foundation Trust and UCL Institute of Ophthalmology, London, UK

**Keywords:** Biologics, Anti-TNF, Uveitis, Optic neuritis, Ocular adverse event, Inflammatory eye disease

## Abstract

The recent introduction of biological agents has revolutionized the treatment of chronic immune-inflammatory diseases; however, this new therapy did not come without significant side effects.

Through large controlled studies indicating decrease in the number of uveitis flares, the role of TNF inhibitors therapy for non-infectious uveitis gained more ground. Paradoxically to its therapeutic effect, there are reports associating these drugs with the onset or recurrence of inflammatory eye disease.

A number of studies have suggested possible roles for anti-TNF-α agents in precipitating or worsening an underlying inflammatory process, including the hypothesis of a disequilibrium in cytokine balance, but to date the mechanisms responsible for these adverse events are not fully understood.

A PubMed literature search was performed using the following terms: ophthalmic complication, uveitis, inflammatory eye disease, optic neuritis, neuropathy, adverse events, anti-TNF, TNF alpha inhibitor, infliximab, etanercept, adalimumab, golimumab, certolizumab, and biologics. The data presented in this study was mainly derived from the use of TNF inhibitors in rheumatology, essentially because these drugs have been used for a longer period in this medical field.

Many of the ocular adverse events reported on this review may be considered a paradoxical effect of anti-TNF therapy. We found a variety of data associating new onset of uveitis with anti-TNF therapy for rheumatic conditions, predominantly under etanercept.

In conclusion, although there is increasing data on ocular adverse events, it remains to be seen whether the suggested link between TNF inhibitors and the onset of ocular inflammation is substantiated by more quality data. Nevertheless, the awareness of potential treatment side effects with anti-TNF should be highlighted.

## Background

Autoimmune inflammatory diseases affect approximately 7.6–9.4% of the world population, especially among the young and middle-aged women. These diseases are on the leading causes of mortality all around the world and are frequently accompanied by severe and chronic morbidity [1].

For the last two decades, the introduction of biological agents has revolutionized the treatment of chronic immune-inflammatory diseases, such as chronic inflammatory rheumatic diseases, and, more recently, uveitis [2]. Although clinically and physiologically different, these diseases all have tumor necrosis factor (TNF) as an important pro-inflammatory cytokine implicated in the pathogenesis and tend to respond positively to drugs targeting it [3].

Anti-TNF drugs are usually well tolerated; however, with the increase of biological therapies, a variety of adverse effects has emerged through mechanisms that are not fully understood. The main adverse event is infusion or injection site reactions, but they are typically manageable. Less common, although potentially more severe, complications include reactivation of tuberculosis and increased incidence of other infections; cytopenias, or, rarely, aplastic anemia; increased risk of malignancy; worsening of congestive cardiac failure; hepatotoxicity; and exacerbation or development of demyelinating disease [4]. Paradoxical inflammatory effects of TNF-α inhibitors have also been noted and include exacerbation or initiation of drug-induced autoimmune diseases, such as psoriasis, systemic lupus erythematosus, vasculitis, and uveitis [5].

In this review, we are going to focus on a less documented aspect of this therapy: ocular adverse events. These range from a common conjunctivitis to endophthalmitis and can potentially be sight-threatening, highlightening the relevance of closely monitoring ocular manifestations in patients treated with anti-TNF-α agents.

It is important to emphasize that when assessing safety profile, the results obtained differ depending on the underlying disease, which may play a crucial role in the rate, type, and severity of adverse events [6].

## Main text

### Aim

The aim of this study was to review the current evidence on ocular adverse events of anti-TNF agents.

### Methods

A literature search was performed in PubMed. The search was focused on the most recent literature but included all articles published after 1990. The keywords ophthalmic complication, uveitis, inflammatory eye disease, optic neuritis, neuropathy, adverse events, anti-TNF, TNF alpha inhibitor, infliximab, etanercept, adalimumab, golimumab, certolizumab, and biologics were used for this search. For this review, mainly studies conducted in medical fields outside ophthalmology were included. Because TNF inhibitors were first implemented in other specialties (e.g., rheumatology), large series of patients have been treated already for a very long time, and a higher quality of evidence can be found in these fields’ literature. References from the selected manuscripts were searched for additional relevant studies.

### Anti-TNF agents

TNF-α is a 26 kDa homotrimeric transmembrane protein that is expressed on the surface of macrophages, T-lymphocytes, natural killer cells, smooth muscle cells, and fibroblasts [7]. In the normal body, TNF has an integral role in mounting the inflammatory response against invading pathogens [8], but in immune-mediated disease, high concentrations of TNF induce excessive systemic inflammation and organ injury via direct pathogenic effects, production of other inflammatory mediators, apoptosis, and tissue destruction [9]. The reduction of TNF-α levels by anti-TNF agents leads to reduced chronic pathologic inflammatory responses [10], markedly improving the outcome of autoimmune inflammatory diseases [11].

This type of therapy includes medications structurally composed of monoclonal antibodies, their Fab portions, or even antibody fusion proteins, which may target cytokines and membrane receptors [12, 13]. There are currently two types of anti-TNF therapies: monoclonal antibodies and soluble receptors and six different drugs (Table 1) [14].

**Table 1:** Monoclonal antibodies

Drug	Etanercept (Enbrel)	Infliximab (Remicade)	Adalimumab (Humira)	Golimumab (Simponi, Simponi Aria)	Certolizumab (Cimzia)
Structure and mechanism of action	TNFR2 ectodomain fused to IgG1 binds TNF-α and TNF-β	Chimeric murine-human IgG1 that binds TNF-α	Fully humanized IgG1 that binds TNF-α	Fully humanized IgG1 that binds TNF-α	Humanized PEGylated Fab that binds TNF-α
Indications	RA, psoriatic arthritis, plaque psoriasis, ankylosing spondylitis, and juvenile idiopathic arthritis	RA, psoriatic arthritis, plaque psoriasis, ankylosing spondylitis, idiopathic pulmonary fibrosis, ulcerative colitis, and Crohn’s disease	RA, psoriatic arthritis, ankylosing spondylitis, spondyloarthritis, juvenile idiopathic arthritis, uveitis (intermediate, posterior, panuveitis), plaque psoriasis, hidradenitis suppurativa, ulcerative colitis, and Crohn’s disease	RA, psoriatic arthritis, ankylosing spondylitis, and ulcerative colitis	RA, psoriatic arthritis, ankylosing spondylitis, spondyloarthritis, and Crohn’s disease
Reported ocular side effects	Uveitis, scleritis, ON, and ocular myositis	Uveitis, ON, and endophthalmitis	Uveitis, ON, endophthalmitis, corneal infiltrates, retinal toxicity, and ophthalmoplegia	ON	ON

### Anti-TNF therapy in ophthalmology

Uveitis is a common, sight-threatening ocular disease, broadly characterized as the inflammation of the uvea. It can have both infectious and noninfectious origin and leads to 5–10% of visual impairment worldwide [15], being considered the third leading cause of preventable blindness in the USA [16].

The current standard of treatment for centers not specialized in uveitis relies on corticosteroid therapy to eliminate inflammation [16]. While both topical and systemic steroids are acutely effective, long-term use of these drugs leads to serious adverse effects, which propelled the search for alternative therapies.

TNF inhibitors began to emerge as a possible therapy for uveitis when a number of large controlled studies indicated that patients with immune-mediated conditions treated with anti-TNF-α agents had a significant decrease in the number of uveitis flares [17]. The results were more obvious with infliximab and adalimumab, compared to etanercept [17].

The VISUAL I and II trials evidenced the ability of adalimumab to reduce the number of uveitis flares compared with placebo (28% and 20% for VISUAL I and II, respectively) [18, 19] and enabled corticosteroid reduction over an 80-week period. These findings were a turning point and contributed to the approval of adalimumab by the FDA and EMA for non-infectious intermediate, posterior, and panuveitis in adults and children older than 2 years of age. They also were pivotal for the approval by NICE in the UK.

A recent systematic review found that adalimumab reduced the risk of treatment failure by 43–75% and might be a promising choice in reducing inflammatory activity, gaining VA, and sparing corticosteroid use in non-infectious uveitis [20].

Although the remaining anti-TNF therapies are considered off-label for uveitis, they are still used to control ocular inflammation associated with systemic diseases [21, 22] and are specially effective in Behçet’s disease [23, 24] and juvenile idiopathic arthritis [25]. Experience with golimumab [26] and certolizumab [27] to treat ocular inflammatory disease is limited but encouraging.

### Ocular adverse events

Because biological TNF-α antagonists have become prominent agents in the management of patients with chronic immune-mediated diseases and their use has increased over the past decade, several adverse events related to its use have been reported, including ocular ones [4].

Paradoxically to its therapeutic effect, there are reports associating TNF inhibitors with the onset or recurrence of inflammatory eye disease, particularly with etanercept [28]. Most cases consist of anterior uveitis (AU), although the induction of intermediate uveitis [29], posterior uveitis [30], scleritis [31], and even orbital myositis [32] has been reported. Other ocular adverse events include blepharitis and ectropion [33], optic neuritis [28], visual impairment [34], diplopia [34–36], endophthalmitis [37, 38], and “sarcoid-like granulomatosis” [39], panuveitis [40], or retinal vasculitis [41].

The role of anti-TNF agents in the pathogenesis of ocular adverse events when used for treatment of diseases in which ocular involvement is recognized as part of the clinical spectrum is controversial. New onset uveitis and development of uveitis in the absence of articular activity after initiation of treatment [28, 32, 42, 43] suggest differing etiopathogenic pathways in ocular and articular inflammation. In contrast, the role of anti-TNF agents in diseases in which some forms of ocular involvement are not recognized as part of the clinical spectrum of the disease, such as RA, seems clearer.

It has been hypothesized [44] that uveitis may occur in isolated patients with rheumatoid arthritis (RA) treated with anti-TNF-α, and the dosages could control the joint manifestations but are inadequate to control uveitis [17] or that a non-detected infectious cause may explain uveitis in some anti-TNF-treated patients [45]. The role of anti-TNF agents in the induction of inflammatory ocular disease (IOD) was also contested by Wendling et al. [29] in a review of 152 cases that reported the resolution of uveitis in most cases even those in which the anti-TNF agent was continued, suggesting a weak impact of these drugs.

However, the increasing evidence associating these therapies to a paradoxical adverse event in ocular inflammation strongly points toward a true cause-effect relation. Furthermore, in many cases, uveitis resolved after treatment withdrawal [46], and re-challenge led to recurrence of the disease [28, 47–49]

### Mechanisms of immunogenicity

The mechanisms whereby TNF-α inhibitors produce paradoxical inflammatory effects are unclear. One hypothesis involves the inverse and interdependent relationship between TNF-α and interferon levels (both α and γ) and the role of increased interferon in the development of autoimmune diseases [50]. Others have suggested that TNF-α inhibition may directly or indirectly increase the rate of infection by organisms implicated in noncaseating granuloma formation [39]. Lastly, it could be dose-related; it is possible that some paradoxical ocular adverse events could be controlled with a high-dose regimen compared to the standard dose given for inflammatory rheumatic disease [5].

In the database of spontaneously reported uveitis under anti-TNF therapy in the USA [28], TNF-α soluble receptor (namely etanercept) was found to be associated with the majority of cases compared to monoclonal antibodies (43 with etanercept, 14 with infliximab, and 2 with adalimumab). After normalizing for the estimated number of patients treated with each medication, etanercept was still associated with a greater number of uveitis cases (*P* < 0.01), and this was again the case after exclusion of patients whose underlying disease was associated with uveitis. In a French study [29] gathering 153 cases, 84% were related to etanercept, suggesting the involvement of differential immunological properties in these two classes of drugs.

There are several hypothesis trying to explain this difference. Regarding pharmacokinetics [51], etanercept has a shorter half-life and volume of distribution and faster clearance than adalimumab and infliximab. Evidence suggests that infliximab is the only one that can induce apoptosis of activated macrophages and T-cells, therefore, theoretically, having a greater impact on controlling immune response [52]. Furthermore, infliximab and adalimumab can shut down both TNFRp55- and TNFRp75-mediated events, while etanercept exerts effect only on TNFRp55 [53]. Differences in reverse signaling and cytokine modulation have also been brought to light [54]. Some authors hypothesized that etanercept has an upregulatory effect over T-cell cytokine responses in contrast to the downregulatory effect of infliximab [55–57]. Lastly, being a soluble receptor, etanercept might be able to extend the half-life of intraocular TNF-α stimulating uveitis until the receptor-ligand complex is removed from the eye [57].

### Results

#### Etanercept

Etanercept (Enbrel®, Amgen Wyeth, Immunex Corporation, Thousand Oaks, CA, USA) was the first anti-TNF-a approved by the FDA in 1998. It is a humanized, recombinant dimeric fusion of a human Fc molecule and two p75 TNF receptors. Etanercept prevents binding of TNF-α and TNF-β to cell surface TNF receptors by binding these circulating factors. Unlike other agents within its class, etanercept does not induce lysis of TNF-producing cells.

##### Uveitis

A registry-based study in the USA found 43 cases of uveitis associated with etanercept [28]. Other studies also showed this association [30, 49]. Compared to the other anti-TNF-α, drugs etanercept had a higher association with uveitis.

The possible reasons for this phenomenon have been previously discussed in this review. One possible explanation is that inhibition with infliximab shuts down both TNFRp55- and TNFRp75-mediated events, whereas etanercept leaves TNFRp75-mediated signaling at least partially intact [53]. Another publication [58] mentions that infliximab maintains a stable complex with sTNF, whereas etanercept does not and that the capacity of soluble p75 receptor (etanercept) to efficiently bind and then release TNF may serve to prolong the circulating half-life of TNF [59].

##### Scleritis

Scleritis is strongly associated with RA, with an overall prevalence of scleritis in patients with RA reported to range from 0.67 to 6.3% [60, 61].

Le Garrec at al [62]. reported three cases of female patients who presented the first episode of unilateral scleritis or acute anterior uveitis while they were treated with etanercept for rheumatologic diseases. Inflammation decreased only when the etanercept was discontinued.

Later, another study [31] described 3 cases of scleritis associated with etanercept use for RA. Ocular inflammation improved after treatment discontinuation, with no other relapses. Re-challenge in one case led to the reappearance of ocular symptoms. When reviewing the literature, the authors found other 8 cases of scleritis associated with the use of etanercept.

##### Optic neuritis (ON)

Various demyelinating disorders have been reported in association with anti-tumor necrosis factor α (TNF-α) agents. Many papers showed the association between optic neuritis and etanercept [28, 63–66]. Patients being treated with anti-TNF-α should be closely monitored for the development of ophthalmological or neurological signs and symptoms.

##### Ocular myositis

Ocular myositis is a rare disorder characterized by inflammation of single or multiple extra-ocular eye muscles presenting with painful diplopia and/or ophthalmoplegia. Two reports of ocular myositis occurring under etanercept treatment for rheumatoid arthritis were found in the literature [67, 68].

#### Infliximab

Infliximab (Remicade®, Janssen Biotech, Inc., Horsham, PA, USA) is a chimeric mouse-human monoclonal antibody that binds and neutralizes both circulating and membrane-bound TNF-α and was the second anti-TNF-α agent to receive FDA approval in 1999.

##### Uveitis

The same registry-based study mentioned with etanercept found 12 cases of uveitis associated with infliximab [28]. A retrospective study found 5 cases of new onset uveitis in patients treated with infliximab [29]. A case report described a patient with ulcerative colitis that likely had a dose-related uveitis with infliximab treatment [69].

##### Optic neuritis

The exact number of cases that present ON as an effect of infliximab or other TNF inhibitors is unknown but seems to be much higher than described in the literature [70]. Many reports state the occurrence of optic neuropathy and neuritis during treatment with infliximab [34, 71–79]. Most present with bilateral and retrobulbar involvement, but different patterns are described.

##### Endophthalmitis

Immunosuppressive disease and therapy are a known risk factor for endophthalmitis [80]. Different case reports documented endophthalmitis during infliximab therapy [38, 81–83]. Jin et al. hypothesized that anti-tumor necrosis factor-α therapy may play a role in the migration of cilia into the globe and the occurrence of endophthalmitis. Scleral thinning due to scleritis [81] is an additional possible mechanism that contributes to the increased risk of endophthalmitis during anti-TNF-α therapy and should be assessed.

#### Adalimumab

Adalimumab (Humira® AbbVie Inc., North Chicago, IL, USA) is a fully humanized monoclonal antibody against TNF-α. Since its approval by the FDA in 2002, it has become one of the most prescribed biologics in the USA [84, 85]. It has 12 approved indications, including non-infectious intermediate, posterior, and panuveitis. To date, treatment with adalimumab for uveitis is still systemic; nevertheless, studies have been made in the direction of producing a more local therapy using intravitreal injections [86–90]. Although most studies demonstrated safety, it is still a controversial topic, and further research is needed to make this method feasible in the future.

##### Uveitis

Although in the registry based study by Lim et al. [28], there were less cases of uveitis during adalimumab therapy (2 cases); then, the other anti-TNF-α, there was no significant difference between adalimumab and infliximab (*p* > 0.5, OR = 1.6). A report by Seve et al. [41] described a woman with RA, on adalimumab for 3 years, who developed bilateral sarcoid-related panuveitis with granulomatous keratic precipitates, periphlebitis, and peripheral multifocal choroiditis.

##### Corneal infiltrates

We found a case report [91] of a woman with Crohn’s disease presenting with bilateral peripheral corneal infiltrates with characteristic features of immune infiltrates 36 h after an adalimumab injection. Symptoms and infiltrates regressed after topical corticosteroid therapy but recurred after each adalimumab injection over the following weeks.

##### Optic neuritis/neuropathy

Once more in the study by Lim et al. [28], the authors found less cases of optic neuritis during adalimumab therapy (4 cases compared to 50 with etanercept and 40 with infliximab). Other authors also reported cases of ON related to adalimumab therapy [92–96].

##### Retinal toxicity

A study evaluating the ocular toxicity of escalating doses of intravitreous adalimumab (Humira) in the rabbit eye [87] found that administration of greater doses was associated with inflammatory reaction and retinal necrosis. We found one report of retinal necrosis following adalimumab therapy in humans [97]. It is possible that this case actually represented a viral retinitis, possibly VZV-related. Another study [98] reported a case of diffuse bilateral retinopathy, detected only by electrophysiology tests, in a woman 1 month after beginning treatment with adalimumab for Crohn’s disease.

##### Endophthalmitis

Only one case report of endophthalmitis associated with adalimumab treatment was found [37]. This report is consistent with the literature on *P. acnes*, since it may induce pathologic reactions in compromised patients and cause endophthalmitis [99].

##### Ophthalmoplegia

In the literature, there is one case report of internuclear ophthalmoplegia in a woman with history of Crohn’s disease who developed facial numbness, blurred vision, and diplopia on right gaze [100].

#### Golimumab and certolizumab

Golimumab (Simponi® and Simponi Aria®, Janssen Biotech, Inc., Horsham, PA, USA) is a fully human monoclonal antibody and was approved by the FDA in 2009.

Certolizumab pegol (Cimzia®, UCB, Inc., Smyrna, GA, USA) is approved for the treatment of moderate to severe rheumatoid arthritis, psoriatic arthritis, and Crohn’s disease. Unlike fellow anti-TNF monoclonal antibodies, this agent is a polyethylene glycolated Fab fragment.

As these are newer types of anti-TNF-α, there is still very little evidence on ocular side effects for both. In the SABER study [101], when reviewing the National Registry of Drug Induced Ocular Side Effects, the authors found 5 cases of ON occurring in association with golimumab or certolizumab. In the same study, cases were most numerous with etanercept (*n* = 169), with fewer reported with infliximab (*n* = 122) and adalimumab (*n* = 55).

### Biosimilars

The spectrum of TNF blockers is constantly expanding. Since the expiration of the patent for infliximab in 2015, a number of biosimilars have obtained EMA and FDA approval and are used in clinical practice. In 2017, biosimilars for adalimumab also became available.

Real-world clinical practice experience and data on these novel drugs are still limited; however, a systematic review showed that biosimilar has a comparable safety and efficacy spectrum to the originating drugs [102]. To this matter, ocular adverse events associated with the original anti-TNF should be closely monitored in therapy with biosimilars. In ophthalmology, a recent study demonstrated that the switch from the original anti-TNF-α to the corresponding biosimilars maintained clinical efficacy in patients with non-infectious uveitis [103].

### Discussion

Although anti-TNF biopharmaceuticals have emerged as a major evolution in the treatment of immune-mediated diseases, the decision to start a patient on anti-TNF therapy has to take into account potentially serious side effects.

Anti-TNF-α is being increasingly used to treat ocular inflammation, and adalimumab and infliximab have the greatest body of data supporting their use in non-infectious uveitis (NIU). These agents are considered appropriate therapy for most forms of NIU but may be considered first-line therapy for uveitis associated with Behçet’s disease and juvenile idiopathic arthritis [104]. Nevertheless, serious potential side effects, such as risk for opportunistic infections, reactivation of latent tuberculosis, and malignancy, may limit their use in ophthalmology.

In addition, a wide range of paradoxical adverse events (PAEs) have been reported [5]. A number of studies have suggested possible roles for anti-TNF-α agents and TNF-α in precipitating or worsening an underlying inflammatory process [105–108]. Among the different theories that have been proposed to explain PAEs, a disequilibrium in cytokine balance is probably the most realistic hypothesis [5]. Multiple immunological pathways are involved in chronic immune-mediated diseases; by adding biological agents, the cytokine milieu is modified, promoting new pathways that create a favorable immunological context for PAE.

Under these circumstances, many of the ocular adverse events reported on this review may be regarded as a paradoxical effect of anti-TNF therapy, particularly uveitis. In this review, we found ample data associating new onset of uveitis with anti-TNF therapy for rheumatic conditions, predominantly in patients with spondyloarthritis and under etanercept [28, 29]. When facing a case of uveitis related to anti-TNF-α, it is important to consider other etiologies associated with the use of these agents, since this type of therapy is associated with increased risk of infections, particularly granulomatous ones [109]. Additionally, inflammatory eye diseases may occur as sign of the underlying systemic disease; hence, in this review, we emphasized cases with dose-related response, temporal relation, positive re-challenge, or new onset disease.

Although awareness of potential treatment side effects should be highlighted, it remains to be seen whether the suggested link between TNF inhibitors and the onset of uveitis is substantiated by more quality data. We gathered mainly case reports and retrospective studies which, considering the broad spectrum of uveitis causes and its potential link to the underlying auto-immune diseases treated with anti-TNF-α, can lead to confounding errors. Future large, prospective, post-marketing studies including patients treated with conventional therapies as controls may be useful in order to evaluate the real risk of patients receiving biological therapies developing uveitis.

Another ocular adverse effect that was found to be consistently associated with anti-TNF-α therapy is optic neuritis. The relationship between anti-TNF-α and induction of demyelinating diseases remains unclear, but in a number of cases the chronology of clinical events is suggestive. A randomized, placebo-controlled study of 168 patients with MS (excluding patients with rapidly progressive MS) treated with lenercept found an increase in the frequency of exacerbations and a trend toward more severe exacerbations when compared with the placebo [110].

Optic neuritis is a serious complication that may lead to irreversible vision loss; therefore, patients being treated with a TNF-α antagonist should be closely monitored for the development of ophthalmological or neurological signs and symptoms. Clinical presentation, serological and radiographic findings, and quick responses to discontinuation of potentially offending drugs suggest ON associated with an anti-TNF agent [63, 65, 71].

If diagnosed, treatment for ON, including pulse steroids followed by oral steroids, should be started. In a literature search, nine out of fifteen patients experienced complete resolution after treatment [65]. Additionally, careful consideration should be done to avoid anti-TNF therapies in patients with a history of demyelinating disease.

In a French national survey [34], the authors found that the disease might persist despite treatment discontinuation, suggesting that anti-TNF-α could trigger the demyelinating process, which further evolves independently. Assessing the same topic, a large US multi-institutional cohort study (SABER) [101] examined ON incidence in patients starting biologic therapy with anti-TNF agents. The authors sensitivity analyses suggest ON rates to be similar in patients who started anti-TNF therapies compared to similarly affected patients who started non-biologic immunosuppressive therapies.

Treatment strategies for ocular adverse events dictate discontinuation of the offending drug. Substitution for steroids is a good option [65–67, 71]; however, this may be a short-term solution in many cases. Another possibility that was considered effective in the literature was switching to other immunosuppressive drugs [63, 96, 111] or other anti-TNF agent classes [30]; however, relapse after introduction of another anti-TNF may occur [34]. Finally, if discontinuation is not an option, pre-treatment with steroids before anti-TNF infusion has been described with positive results [69].

## Conclusion

Although there is increasing data on ocular adverse events associated with anti-TNF-α therapy, most available information comes from uncontrolled studies, and therefore solid conclusions are not possible. In spite of the limited quality of the available data, this review is important to gather the current information and shed light on the possibility of potential adverse and paradoxical effects of anti-TNF agents that rheumatologists and ophthalmologists should be aware.

Diagnosis and treatment must be co-managed by rheumatologists and ophthalmologists. In some cases, cessation of the drug is mandatory due to persisting and/or recurrent eye disease.

Finally, our findings do not alter the current indications for the use of biologics but rather recommend a close surveillance of ocular status in patients using anti-TNF and open the discussion about the need of better quality evidence on this matter.

## Data Availability

Not applicable

## Abbreviations

AU: Anterior uveitis
IgG: Immunoglobulin G
IOD: Inflammatory ocular disease
NIU: Non-infectious uveitis
ON: Optic neuritis
PAE: Paradoxical adverse events
RA: Rheumatoid arthritis
TNF: Tumor necrosis factor
